# Specialty training for the retention of Malawian doctors: A cost-effectiveness analysis

**DOI:** 10.1016/j.socscimed.2017.10.012

**Published:** 2017-12

**Authors:** Kate L. Mandeville, Kara Hanson, Adamson S. Muula, Titha Dzowela, Godwin Ulaya, Mylène Lagarde

**Affiliations:** a Department of Global Health and Development, London School of Hygiene and Tropical Medicine, London, United Kingdom; b Department of Public Health, School of Public Health and Family Medicine, College of Medicine, University of Malawi, Blantyre, Malawi; c Department of Clinical Services, Ministry of Health, Lilongwe, Malawi; d Johns Hopkins Project, Blantyre, Malawi

**Keywords:** Malawi, Cost-effectiveness analysis, Discrete choice experiment, Human resources for health, Physicians, Education medical, Specialization

## Abstract

Few medical schools and sustained emigration have led to low numbers of doctors in many sub-Saharan African countries. The opportunity to undertake specialty training has been shown to be particularly important in retaining doctors. Yet limited training capacity means that doctors are often sent to other countries to specialise, increasing the risk that they may not return. Expanding domestic training, however, may be constrained by the reluctance of doctors to accept training in their home country. We modelled different policy options in an example country, Malawi, to examine the cost-effectiveness of expanding specialty training to retain doctors in sub-Saharan Africa. We designed a Markov model of the physician labour market in Malawi, incorporating data from graduate tracing studies in 2006 and 2012, a 2013 discrete choice experiment on 148 Malawian doctors and 2015 cost data. A government perspective was taken with a time horizon of 40 years. Expanded specialty training in Malawi or South Africa with increasing mandatory service before training was compared against baseline conditions. The outcome measures were cost per doctor-year and cost per specialist-year spent working in the Malawian public sector. Expanding specialty training in Malawi is more cost-effective than training outside Malawi. At least two years of mandatory service would be more cost-effective, with five years adding the most value in terms of doctor-years. After 40 years of expanded specialty training in Malawi, the medical workforce would be over fifty percent larger with over six times the number of specialists compared to current trends. However, the government would need to be willing to pay at least 3.5 times more per doctor-year for a 5% increase and a third more per specialist-year for a four-fold increase. Greater returns are possible from doctors with more flexible training preferences. Sustained funding of specialty training may improve retention in sub-Saharan Africa.

## Highlights

• Innovative cost-effectiveness analysis of specialty training to retain doctors.
• We designed a Markov model of the labour market for doctors in Malawi.
• Expanding domestic training is more cost-effective than funding regional training.
• At least two years of mandatory service before training is more cost-effective.
• Sustained funding of specialty training may improve retention in sub-Saharan Africa.

## 1 Introduction

Low production and high emigration have led to few doctors in many sub-Saharan African countries, impeding the delivery of essential health services and response to new health threats (Sidibé and Campbell, 2015, World Health Organization, 2006, World Health Organization, 2015). Retaining doctors in their country of training, therefore, has been the focus of recent policy efforts. Out of possible incentives, the opportunity to specialise has been found to be particularly important to doctors (George et al., 2007, Mandeville et al., 2014a, Willis-Shattuck et al., 2008). Yet the specialist workforce is also small in many of these countries, constraining domestic training capacity and forcing countries to send doctors to other countries to specialise (Mullan et al., 2011). The opportunity to train in more advanced health systems is often popular with doctors, but augments the risk of emigration. This presents a dilemma for policymakers: sending doctors to train in other countries is likely to increase retention of doctors in the short-term, but may produce specialists more likely to emigrate in the long-term. Stipulating a mandatory period of work before entry to specialty training would ensure better value from this investment, but delay the production of much-needed specialists. Expanding domestic training may protect against emigration of specialists through the strengthening of personal and professional ties, but may not be accepted by doctors if external training is a possibility. Finally, many countries will be unable to reduce their dependence on foreign-trained specialists without expansion of specialty training, yet specialists are costlier to produce and employ than generalist doctors.

To aid decision-making in this area, a formal comparison of the costs and effects of different policy options would be appropriate. Such economic evaluations, however, have been rarely employed in health workforce policy to date. Only one study, to our knowledge, has used decision analytical modelling to evaluate the cost-effectiveness of different health workforce policies (Lagarde et al., 2012). One key theoretical issue that may have limited empirical studies is this area is the value of health workers. Despite numerous attempts to correlate health worker density with health service or population health outcomes, the relationship between population health and the availability of health workers remains ill-defined (Anand and Barnighausen, 2004, Anand and Barnighausen, 2007, Castillo-Laborde, 2011, Cochrane et al., 1997, Hertz et al., 1994, Kim and Moody, 1992, Robinson and Wharrad, 2000, Robinson and Wharrad, 2001, Speybroeck et al., 2006). In contrast to clinical or public health interventions, therefore, it is more difficult to express the effects of health workforce policies in outcomes such as life-years gained or mortality averted. Yet for national decision-makers seeking to make the best use of limited budgets with the existing health workforce, the most important outcome in the timeframe of the decision is maximising clinical contact over the population, with the most natural unit being clinician-time. Indeed, the study mentioned above uses the outcome of ‘rural nurse-years’ (the number of years worked by a nurse in a rural health facility) to evaluate different policies to recruit and retain nurses in rural areas over their working life (Lagarde et al., 2012). As more definitive evidence becomes available on the relationship between density of health workers and population health, it may be possible to move to more downstream outcomes.

Such dynamic modelling and economic evaluation of different policy options are particularly well suited to health workforce decisions in low- and middle-income countries (LMIC) (Lagarde and Cairns, 2012, Mandeville et al., 2016a). For many cadres of health worker, the lag between training investment and labour production means that costs and effects can only be fully evaluated over the long-term. Training and retaining health workers also consumes considerable resources from limited budgets. For example, out of its 2014/2015 health budget, the Malawian government spent around 4% on training and 36% on salaries (Ministry of Health data). Decisions on health workforce policy are therefore high value, yet usually made in a low-information environment. Indeed, another constraint on the use of economic evaluations in this area is the lack of evidence on the effectiveness of different policies, compounded by a general paucity of data on health workforce dynamics in LMIC for model inputs. Recognition of this issue has led to increasing use of discrete choice experiments, a survey method that quantifies the preferences of health workers by asking them to make choices between jobs offering different conditions (Mandeville et al., 2014a). These stated preferences can then be used as a guide to the uptake and thus effectiveness of different policies in economic evaluations (Terris-Prestholt et al., 2016) Although revealed preferences derived from experimental studies would be ideal, these types of studies are difficult to undertake with centralised workforces. Moreover, observational data of actual labour market decisions may not accurately reflect preferences where available job options are constrained, as in many LMIC health labour markets. Discrete choice experiments, in contrast, enable comparison of both existing and potential policies and are therefore provide a useful measure of policy effectiveness in this context.

To further the empirical literature in this area, this study models the medical workforce in an example country, Malawi, to assess the cost-effectiveness of different specialty training policy interventions. Following the approach of the previous study (Lagarde et al., 2012), we use the outcome measure of doctor-years, defined as the number of years worked by doctors in the Malawian public sector. We employ the results of a previous discrete choice experiment to estimate the effectiveness of interventions, as well as tracing studies of Malawian medical graduates to estimate transition probabilities (Mandeville et al., 2014b, Mandeville et al., 2016b, Zijlstra and Broadhead, 2007).

## 2 Study setting

In Malawi, which has one doctor for every 53,000 people, a national medical school was established in 1991 (Broadhead and Muula, 2002, Muula and Broadhead, 2001, World Health Organization, 2015). In 2005, concerns over the continuing low numbers of doctors led to a tripling of medical students at the College of Medicine-University of Malawi (COM), but no proportionate increase in the number of specialty training places available (Management Sciences for Health, 2010). The vast majority of medical students and junior doctors still intend to specialise, raising concerns that they may emigrate if training opportunities are not available in Malawi (Mandeville et al., 2012). Indeed, more specialists are needed in Malawi, with many budgeted public sector posts across all specialties vacant. For example, in 2014, there were just ten ophthalmologists in Malawi, seven of whom were Malawian (Schulze Schwering et al., 2014). Limited national capacity for specialty training, however, means that some or all training for certain specialties is undertaken in South Africa and other African countries. Yet historically those doctors who specialise outside Malawi have been far less likely to remain in Malawi (Muula, 2009, Muula and Broadhead, 2001, Zijlstra and Broadhead, 2007). Where training can be provided in Malawi, junior doctors have been reluctant to take this up due to concerns over transferability of training, remuneration during training and the perceived quality of training in a resource-poor setting (Bailey et al., 2012, Sawatsky et al., 2014).

## 3 Methods

**Fig. 1:**
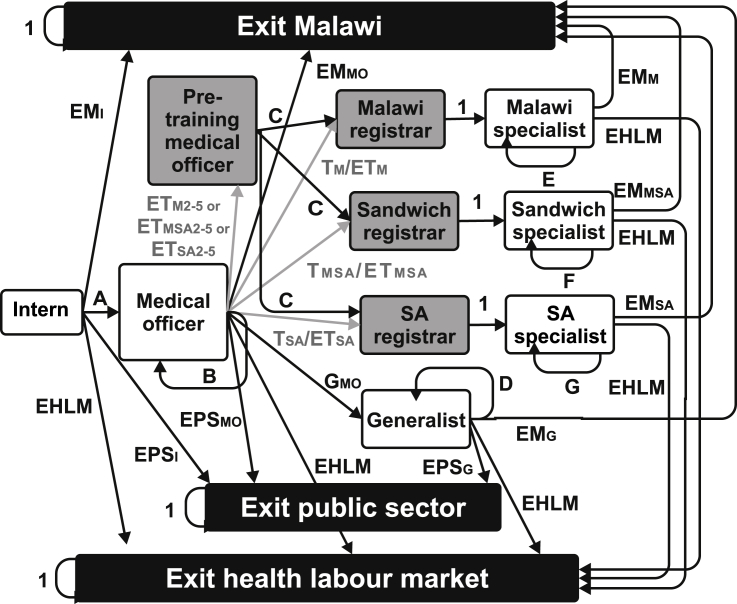
**Model figure**. SA = South Africa; absorbing states in black; tunnel states in grey; all other temporary states in white; transition probabilities shown and explained further in technical appendix; grey lines and text indicate transition probabilities affected by policy interventions; *A* = 1*- EM*_*I*_*-EPS*_*I*_*–EHLM; B* = 1- *ET - T*_*M*_*- T*_*MSA*_*- T*_*SA*_*-EM*_*MO*_*- EPS*_*MO*_*–EHLM; C* = 1 – *EHLM; D* = 1- *EM*_*G*_*- EPS*_*G*_*– EHLM; E* = 1- *EM*_*M*_*–EHLM; F* = 1- *EM*_*MSA*_*–EHLM; G* = 1- *EM*_*SA*_*–EHLM.*

Following Lagarde and Cairns (Lagarde and Cairns, 2012), we used a Markov model to develop a simplified model of the prevailing labour market for Malawian doctors (Fig. 1). In this model, a doctor can be in one of a limited number of mutually exclusive states, which represent jobs in the Malawian public sector or a position outside it. A Markov process was used to model the movement of doctors over their working lifetime. This is essentially a closed hierarchical system, where doctors enter at the most junior level and can only transition to more senior states. At the end of each cycle in the model (set to one year), a doctor can transition to another state.

To identify all relevant costs and consequences, the time horizon is the working life of a doctor in Malawi. The mean age of newly graduated doctors in Malawi is 24 years and the mandatory retirement age for both sexes in the public sector is 55 years (Government of Malawi, 2011, Management Sciences for Health, 2010, Muula and Maseko, 2005). The working life of many doctors, however, has been extended past this age using repeated fixed-term contracts to boost retention (Management Sciences for Health, 2010). Given this higher effective retirement age and likely increases in the mandatory retirement age over the lifetime of new graduates, the time horizon was set as 40 years.

The perspective taken is that of the Malawian government. As the government pays the salaries of Malawian doctors working in rural health facilities run by faith-based organisations, these doctors are also considered to be working in the public sector.

As outlined above, we used the outcome measure of doctor-years, defined as the number of years worked by qualified medical doctors in the Malawian public sector. Not all doctor-years are of the same value, however, and in order to distinguish doctors with higher levels of human capital, we also measured specialist-years: the number of years worked by qualified medical specialists in the Malawian public sector. The baseline scenario represents the current situation in Malawi with limited training places available and estimates the cumulative impact on the outcome measures if there is no change in training policy. The alternatives examine the impact of different policy interventions that expand specialty training. This training varies in location and in the requirement to complete a mandatory service period of two to five years before training (see below).

### 3.1 Description of states

States can be temporary (entered but eventually left) or absorbing (entered but never left). Our model consisted of ten temporary states and three absorbing states that reflect three types of exit from the public sector. The first, “exit Malawi”, encompasses doctors who have left Malawi to work or train outside of government training programmes. In addition, all doctors who have left Malawi for specialty training not funded by the government are assumed to remain outside Malawi. The “exit health labour market” state incorporates retirement, death and doctors who have changed professions or decided not to work. For tractability, we have not modelled doctors who temporarily leave the labour market to raise children. The “exit public sector” includes all doctors working exclusively in private practice or for commercial companies, non-governmental organisations or research/teaching institutions. A matrix of all transition probabilities between states is included in the technical appendix, along with more detail on their estimation and initial values.

Doctors enter the model in the “intern” state. All medical graduates in Malawi have to complete an 18-month internship in the public sector in order to gain full registration with the Medical Council of Malawi (Muula and Maseko, 2005). For simplicity, we have modelled this as a temporary state lasting one cycle.

All doctors then move to the “medical officer” state unless they enter one of the absorbing states. The probabilities of exiting the public sector and outside Malawi from the intern state were informed by a 2012 tracing study of COM graduates from 2006 to 2012 (Mandeville et al., 2014b). The probability that doctors exit the health labour market increases over the time horizon, with values informed by a 2006 tracing study of all COM graduates from 1991 (Zijlstra and Broadhead, 2007).

Once in the medical officer state, doctors remain there unless they exit into one of the absorbing states or enter specialty training. The transition probabilities for exiting Malawi and the public sector were also informed by the 2012 tracing study, with the latter shown to be lower for medical officers than for interns. The probability of transitioning to specialty training in the baseline scenario was informed by MOH and COM administrative data (see technical appendix). The probability of entering specialty training under different policy interventions was informed by results from a discrete choice experiment (see below).

If a policy intervention involves a mandatory service period before training, doctors first enter a “pre-training medical officer” state for the required number of cycles before starting training. This is a tunnel state, i.e. a temporary state that can last a number of cycles but cannot be exited during this period.

Doctors undertaking specialty training are known in Malawi as registrars. There are three registrar states in the model, depending on the location of training. As most registrars training outside Malawi under government funding are sent to South Africa, this country was used in the model. Thus, a “Malawi registrar” trains fully in Malawi, a “South Africa registrar” trains fully in South Africa and a “sandwich registrar” undertakes training split between Malawi and South Africa. The allocation between these states in the baseline scenario was based on current training patterns. All three states are modelled as four-cycle tunnel states, which is the standard length of specialty training programmes for clinical specialties. Only time spent in Malawi counted towards the outcome measures.

All registrars then enter one of three specialist states, depending on the location of their training: “Malawi specialist”, “South Africa specialist” or “sandwich specialist”. Most specialists in Malawi are employed in the public sector even if they undertake dual practice. Therefore we have assumed that no specialist exits the public sector, although they may exit Malawi or the health labour market. We used limited data on the current location of specialists trained in the last ten years to estimate the risk of exiting Malawi for specialists trained in different locations. We anticipated this value to decline over time both within a cohort, as job mobility diminishes in later career (Padarath et al., 2003, Tjadens et al., 2012), and also over cohorts, due a likely “magnet effect”. This reflects the positive influence of a growing specialist workforce on retention of new specialists due to role modelling and greater opportunities for professional interaction (Bailey et al., 2012).

To distinguish medical officers from more senior doctors who have not specialised, we also created a “generalist” state. This represents doctors working in central and district facilities, as well as administrative positions. It also incorporates doctors working as general practitioners, as specialty training in general practice (family medicine) has only recently been established in Malawi. All doctors still in the medical officer state after six years will transition into the generalist state (modelled as a transition probability of one at the end of cycle 7). Although doctors could still seek and enter specialty training in their later working life, it is far less common than in the early career period. Therefore, generalists remain as generalists unless they enter one of the absorbing states. The transition probabilities for exiting Malawi and the public sector for generalists also decline over time, with the initial value equal to those for the medical officer state.

The main structural assumptions of the model are as follows. As in the norm in medical careers, doctors can only progress up the professional hierarchy and cannot return to a more junior position. For simplicity, it is assumed that doctors do not go straight into specialty training on completion of internship, although this does occur *de facto*. Transition to training occurs at the end of one cycle for a cohort rather than throughout the junior doctor period. All registrars are assumed to complete their training within four years without dropping out. All registrars return to Malawi for at least one year of practice as a specialist after completion of training. Junior doctors who do not enter specialty training within seven years of qualifying do not enter it later in their careers. Finally, the “exit public sector” state was modelled as an absorbing rather than temporary state. Repeated movement between sectors is uncommon in Malawi, with health workers reporting strong barriers to returning to the public sector after exit (Muula and Maseko, 2005). This is compounded in a hierarchical cadre such as medicine, where re-entry risks a loss of seniority and/or status. The most flexible period is likely to be early career, yet examination of employment data collected on Malawian junior doctors showed that no doctor had returned to the public sector after a job outside it. Although data were not available to confirm this assumption for the entire medical workforce, there was sufficient justification to model an exit from the public sector as an absorbing state for these purposes. However, barriers to re-entry into the public sector – both for those outside the public sector and emigrant doctors – may lessen over the time horizon, therefore the durability of the absorbing states over the time horizon is also a strong assumption of this model.

### 3.2 Policy interventions

The policy interventions all expand specialty training so that there are sufficient places available for all junior doctors who are willing to take up this training. They differ in the location of this expanded training (all in Malawi, “sandwich” i.e. split between Malawi and South Africa, or all in South Africa), and whether it is combined with a mandatory service period before commencing training of two, three, four or five years in Malawi. These combinations result in 15 alternatives to be compared to the baseline scenario (listed in Table 1).

The effectiveness of each intervention is taken as the uptake of available training places by medical officers. These were based on predictions from a discrete choice experiment that quantified the preferences of junior doctors in Malawi for different types of specialty training (Mandeville et al., 2016b). Junior doctors were asked to choose between two jobs: both guaranteeing a specialty training place, but with training differing in location, the number of years of mandatory service and the specialty itself. As the potential of expanding specialty training as an incentive to retain doctors was the primary focus of this study rather than the choice of specialty, a constant ratio of popular to unpopular specialties (also examined in the study) was used to predict uptake. The predicted uptake rates can be found in the technical appendix and form the transition probabilities between medical officer and registrar (*T**m, Tmsa, Tsa*) or medical officer to medical officer before training (*E**T*_*M2-5,*_ *E**T*_*MSA2-5,*_ *E**T*_*SA2-5*_). Those who opt out of the posts on offer remain in the medical officer state, but are subjected to the background transition probabilities to the absorbing states before transitioning to the generalist state. These uptake rates were assumed to be constant over the time horizon.

### 3.3 Model population

To explore the cumulative impact of different policy scenarios, the model followed 40 successive cohorts of Malawian doctors, with each cohort entering one cycle after the preceding cohort. All cohorts start in the intern state. The number of graduates at COM has increased from 13 in 1992 to 69 in 2014, with annual enrolment averaging 99 over the past five years. The size of the first cohort was therefore set as 100. As medical student numbers are likely to rise further over the next 40 years, each subsequent cohort was expanded by five graduates leading to a final cohort size of 295.

The existing stock of Malawian doctors was also incorporated in the model. We used data from previous tracing studies of Malawian doctors and also the Medical Council of Malawi to inform the distribution of these doctors across model states in cycle 1 (see technical appendix).

### 3.4 Cost estimates

There are two types of cost relevant here: in-service costs and specialty training costs. These incorporate the direct costs of training and employing more doctors and specialists and are summarised below, with further details and all values set out in the technical appendix. Indirect costs such as the administrative burden of policy implementation or increased service costs associated with more specialist medical activity were deemed either negligible compared to the direct costs or too difficult to attribute directly to the policy intervention rather than the general needs of the health service.

The major in-service costs comprise salaries and government pension contributions. The most recent MOH salary scales were used to estimate salaries within each state, combined with a 10% uplift to cover pension contributions. All public sector doctors are also entitled to subsidised accommodation. Data on government rental costs for doctors’ housing were collected in order to estimate an average cost across states. Specialists in Malawi also receive a one-off allowance for a vehicle and monthly allowances for fuel and communication costs. Interns and medical officers are also provided transport to and from work.

The MOH pays for specialty training, whether in Malawi or South Africa. In Malawi, the MOH pays the COM tuition fees for specialty training programmes. In South Africa, the MOH pays the tuition fees of South African universities for each year that Malawian registrars are in South Africa. Four universities have taken Malawian registrars in the past: University of Cape Town, University of the Witwatersrand, University of Pretoria and University of KwaZulu-Natal. The mean of relevant fees for Malawian doctors in specialty training programmes at these universities was used to represent annual tuition fees in South Africa. We also incorporated various allowances paid to registrars in Malawi and South Africa in recent funded training places.

### 3.5 Modelling cost-effectiveness and sensitivity analysis

The model was constructed using Microsoft Excel 2013, following the approach of Briggs et al., and is available as a supplementary file (Briggs et al., 2006). A discount rate of 3% was applied to both costs and effects. A probabilistic sensitivity analysis was carried out to account for uncertainty in the input parameters. Here, the value of each parameter is considered to be random rather than fixed, with an associated probability distribution. The distribution employed for each parameter followed standard practice in health economics (Briggs et al., 2006). 2000 Monte Carlo simulations were used to draw values from these distributions, with the mean used to examine the distribution of doctors across different states in the model, as well as compute the average costs and effects for each intervention. The latter were used to calculate the incremental cost-effectiveness ratio (ICER) for each intervention compared to the baseline scenario, calculated for both doctor-years and specialist-years.

As an intuitive alternative to confidence intervals around ICERs, we calculated cost-effectiveness acceptability frontiers (CEAF) over zero to MWK50 million in increments of MWK100,000. CEAFs identify the optimal option (i.e. that with the highest net monetary benefit) over a range of cost-effectiveness thresholds and then plot the probability that this option is cost-effective over all values. MWK amounts were expressed in international dollars using 2015 data (World Bank, 2016).

### 3.6 Threshold value by outcome measure

Current suggested cost-effectiveness thresholds for LMIC are based on disability-adjusted life-years averted (Tan-Torres Edejer et al., 2003). As outlined above, the outcomes in this analysis are not easily expressed in population health terms. andWe therefore used an alternative strategy to identify the threshold that the Malawian government is currently willing to pay for a doctor- and specialist-year by using the data here to calculate the net present value of the discounted costs of an “average” doctor working for 40 years in the public sector (see technical appendix).

### 3.7 Subgroup analysis

The discrete choice experiment identified four subgroups with substantially different preferences with regard to specialty training (Mandeville et al., 2016b). These were characterised as:

(i) “rich rejecters” (high current salary, frequently refused the hypothetical jobs in the public sector offered to them);
(ii) “money motivated” (greatest preference for salary increases);
(iii) “stubborn specialists” (strong training preferences with little flexibility);
(iv) “pliant patriots” (most flexible training preferences, least disutility from training in Malawi).

As these preferences affected the predicted uptake of training places (see technical appendix), we ran separate analyses for each subgroup to assess the impact of this heterogeneity on the cost-effectiveness of the policy interventions.

### Ethical approval

Ethical approval for this study was obtained from the COM Research and Ethics Committee of the University of Malawi (P.09/11/1129) and the London School of Hygiene and Tropical Medicine (6042).

## 4 Results

### 4.1 Distributions across states

**Fig. 2:**
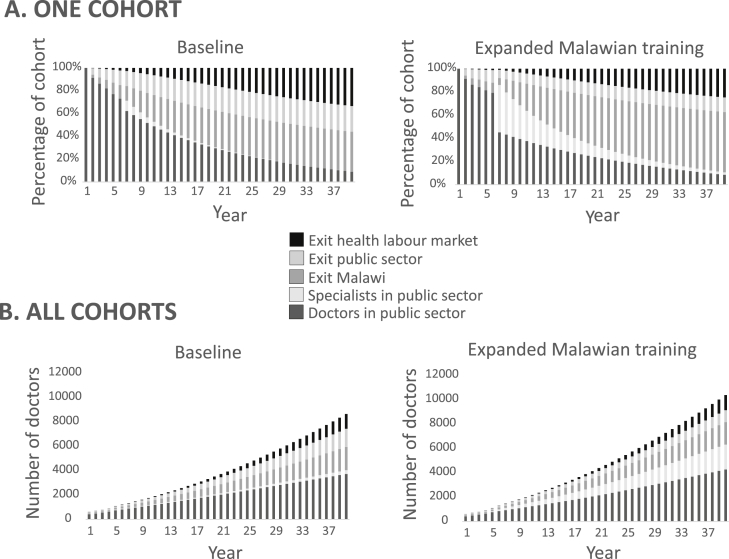
**Distributions across states over time**. A. Distribution of first cohort of doctors across states over time horizon. B. Distribution of all cohorts over time horizon.

The impact of different policy interventions can be seen from the distribution of doctors across model states over the time horizon. Fig. 2 shows the progression of the first cohort of doctors in the baseline compared to an illustrative policy intervention of expanded training in Malawi. At the end of year 6, specialists start to complete their training leading to a rise in their number in the public sector. However, without the magnet effect of a professional community, many of these specialists leave Malawi by the end of the time horizon. The distribution of all doctors in the model (existing stock and all 40 cohorts) is also shown in Fig. 2. With expanded specialty training in Malawi, the medical workforce would be over 50% greater and the number of specialists in Malawi more than six times that of baseline at the end of the time horizon. The number of doctors outside Malawi is roughly equal, however fewer doctors exit the public sector under expanded training compared to baseline.

### 4.2 Average costs and effects

Total doctor-years and specialist-years for each intervention as a proportion of baseline are shown in Table 1, with values and total costs in the technical appendix. Expanding Malawi or sandwich training with or without mandatory service results in greater doctor- and specialist-years than baseline, whereas expanding training in South Africa in any form actually reduces total doctor-years (by over a fifth without any service). The greatest gains in doctor-years (17%) and specialist-years (516%) would come from expanding sandwich training without any service.

**Table 1:** Effects as proportion of baseline for whole population and subgroups.

Policy intervention^a^	Doctor-years as proportion of baseline					Specialist-years as proportion of baseline				
	WP	RR	SS	MM	PP	WP	RR	SS	MM	PP
Expanded Malawi training	1.07	1.02	1.07	1.08	1.12	5.90	2.61	5.49	6.00	8.52
Expanded sandwich training	1.17	1.10	1.17	1.18	1.23	6.16	3.76	5.86	6.22	7.87
Expanded South Africa training	0.78	0.87	0.79	0.76	0.72	5.80	3.76	5.59	6.29	7.03
Expanded Malawi training + 2 years	1.07	1.01	1.07	1.08	1.13	3.96	1.22	3.81	4.01	6.10
Expanded sandwich training + 2 years	1.01	1.00	1.01	1.01	1.01	4.47	1.61	4.46	4.70	6.17
Expanded South Africa training + 2 years	0.89	0.98	0.89	0.87	0.85	4.11	1.58	4.08	4.73	5.29
Expanded Malawi training + 3 years	1.07	1.00	1.07	1.07	1.12	3.22	0.96	3.13	3.26	4.90
Expanded sandwich training + 3 years	1.02	1.01	1.02	1.02	1.03	3.65	1.06	3.68	3.86	5.16
Expanded South Africa training + 3 years	0.93	1.00	0.93	0.91	0.90	3.39	1.11	3.43	3.97	4.38
Expanded Malawi training + 4 years	1.06	1.00	1.06	1.07	1.11	2.63	0.88	2.55	2.67	3.85
Expanded sandwich training + 4 years	1.03	1.01	1.03	1.04	1.05	2.98	0.85	3.02	3.19	4.14
Expanded South Africa training + 4 years	0.96	1.01	0.96	0.95	0.94	2.82	0.87	2.90	3.35	3.57
Expanded Malawi training + 5 years	1.05	1.00	1.05	1.05	1.09	2.10	0.88	2.10	2.15	2.92
Expanded sandwich training + 5 years	1.04	1.00	1.04	1.04	1.05	2.39	0.72	2.50	2.60	3.18
Expanded South Africa training + 5 years	0.99	1.01	0.98	0.98	0.98	2.31	0.81	2.38	2.77	2.77

**Notes:** WP = whole population; RR = rich rejecters; SS = stubborn specialists; MM = money motivated; PP = pliant patriots.

a Years refer to number of years of mandatory service for that intervention.

When total costs are broken down into different cost categories, salaries constitute between 74 and 80% of spending and training costs just 1–2% across most interventions (see technical appendix). The exceptions are interventions without any mandatory service, where training rises to 7–8% and salaries drop to 62–65%. A more substantial cost arising from expanded specialty training is the “perks” provided to qualified specialists, particularly the monthly fuel allowance, running from 7 to 19% of total costs.

### 4.3 Threshold value by outcome measure

Using the available data to calculate the net present value of the discounted costs of both an “average” doctor and a specialist working for 40 years in the public sector, we calculated that the Malawian government currently values a doctor-year at MWK5.5 million (intl. $34,700) and a specialist-year at MWK7.9 million (intl. $50,300).

### 4.4 Cost-effectiveness acceptability frontiers

**Fig. 3:**
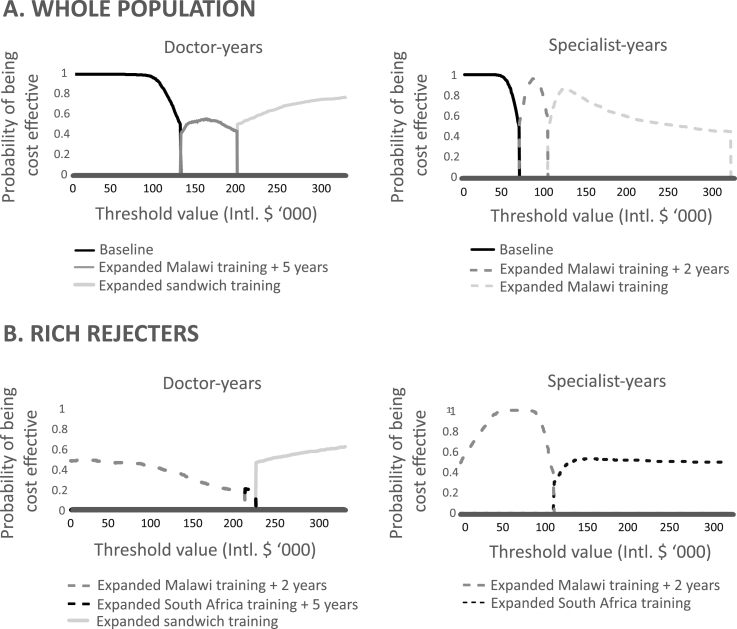
**Cost-effectiveness acceptability frontiers**. A. Cost-effectiveness acceptability frontiers for doctor-years and specialist-years for whole population. B. Cost-effectiveness acceptability frontiers for doctor-years and specialist-years for rich rejecters subgroup.

The incremental effects, costs and ICERs for all interventions for the whole population and by subgroup are provided in the technical appendix. ICERs, however, do not reflect well the uncertainty associated with the cost-effectiveness of an intervention, which are better displayed by CEAFs (Fig. 3). For both doctor- and specialist-years, the optimal option above baseline is expanded training in Malawi with some mandatory service. If the government is willing to pay between MWK 19.8 and 30.1 million (Intl. $126,000 to 191,600) per doctor-year, the optimal option is expanded Malawi training with five years' mandatory service, although there is uncertainty whether this is more cost-effective than expanded sandwich training at this threshold. If the government is willing to pay between MWK 10.5 and 15.7 million (Intl.$66,800 to 99,000) per specialist-year, the optimal option with high certainty is expanded Malawi training with two years’ mandatory service.

The most cost-effective policy intervention to increase doctor-years, expanding Malawian training with five years’ mandatory service, would therefore require the government to be willing to pay three and a half times the current spending on a doctor-year. To expand the specialist workforce most cost-effectively, the government would need to be willing to pay a third more than current spending on a specialist-year.

### 4.5 Subgroup analysis

The only two subgroups that showed substantial differences to the whole population were the pliant patriots and the rich rejecters. Pliant patriots had the highest uptake rates of training for all locations and all lengths of mandatory service. Expanded training in Malawi would lead to a greater number of doctor- and specialist-years than for the whole population with any length of mandatory service, including a potential 8.5-fold increase in specialist-years with no service requirement (Table 1). The optimal options are the same as for the whole population, although the greater uptake of training places leads to slightly higher thresholds of MWK 20.5 million (Intl. $ 130,500) per doctor-year and MWK 11.5 million (Intl. $73,200) per specialist-year. For rich rejecters, the optimal option is expanded Malawi training with two years’ mandatory service, which is cost-saving over baseline with an ICER of MWK -43.4 million (Int.$276,500) per doctor-year and MWK -21.2 million (Int.$134,900) per specialist-year.

## 5 Discussion

The findings of this economic evaluation show that the Malawian government could obtain higher returns on their investment in medical education by expanding specialty training in Malawi. Funding more doctors to train exclusively outside Malawi will lead to less return than current policies. At least two years of mandatory service in the public sector before training in Malawi would be more cost-effective, with five years adding the most value in terms of doctor-years. For doctors who are likely to leave the public sector after internship, two years of mandatory service would be cost-saving over current policies. After 40 years of expanded training in Malawi, the medical workforce would be over 50% larger and there would be over six times the number of specialists compared to current trends. These policies, however, would be more costly than current government spending for relatively modest gains in doctor-years. The government would need to be willing to pay at least 3.5 times more per doctor-year for a 5% increase in total doctor-years and a third more per specialist-year for a four-fold increase. Greater returns would be possible from those doctors with more flexible preferences.

This is the first evaluation, to our knowledge, of the cost-effectiveness of specialty training for retaining doctors in LMIC. By including two outcome measures, this study recognises the often divergent objectives in health workforce policy and provides more information for stakeholders. This study demonstrates the impact of predictable financing of specialty training on Malawi's specialist workforce in comparison to the current *ad hoc* funding. It provides a realistic assessment of the costs of these policies compared to current government spending. Finally, while mandatory service has been considered in Malawi in light of the heavy subsidy of undergraduate medical training (Muula and Maseko, 2005), this study is the first to establish its cost-effectiveness. Whilst compulsory service is often defaulted on when linked to financial incentives (Bärnighausen and Bloom, 2009), its stipulation as an entry requirement to specialty training is more feasible and common in other countries (Frehywot et al., 2010).

The cost-effectiveness analysis by Lagarde et al. also used a discrete choice experiment to establish the effectiveness of different incentive policies, but its focus was on the recruitment and retention of South African nurses to rural areas (Lagarde et al., 2012). Although the different scope limits any comparison with the results obtained here, this study did find that offering nurses study leave to specialise was more cost-effective than financial incentives alone. Overall, an “upstream” intervention of recruiting more nurses from rural areas was the most cost-effective option. This echoes our findings in the pliant patriots subgroup, who were more likely to accept specialty training in Malawi than the overall population - although this greater acceptance led to a slightly higher cost-effectiveness threshold. Junior doctors in this subgroup required the least compensation to train all in Malawi and were the only subgroup for whom training outside Africa did not significantly influence their choices (Mandeville et al., 2016b). They also tended to be older than their peers and earned less on average, so these more flexible preferences may reflect an increasing pragmatism about any training places on offer. Their acceptance of training in Malawi, however, may be shared by more junior doctors as the COM training programmes become better established. Methodologically, our study substantially extends this study through more complex modelling, incorporating the existing stock of Malawian doctors, representing a magnet effect through cohort-dependent transition probabilities, and distinguishing health workers with different levels of human capital. We also provide the model file to aid other researchers.

It is important to recognise the scale of the challenge facing the health workforce in Malawi. Our model, which provides the most robust forecast of Malawi's medical workforce to date, shows that the optimal option would expand the number of doctors by 50% by the end of the time period. Yet Malawi's population is predicted to increase by 170% over the next 40 years (United Nations, 2015). Any gains in increased production and retention of doctors will be quickly outweighed by population growth, unless consideration is given to complementary policies such as expansion in undergraduate training or other cadres such as clinical officers. Future cost-effectiveness analyses examining the optimal mix of health workers would be useful in this regard.

There are several limitations to this analysis. First, we took a government rather than a societal perspective, thus disregarding any welfare produced by doctors working outside the public sector. The stock of doctors in Malawi is so small that clearly the labour of all doctors is of social value, yet the largest disease burden exists in rural areas with 80% of the population. As the vast majority of facilities in rural areas are public rather than private, a government perspective captures the doctor-years holding the most marginal value for Malawi's population. Although the availability of several tracing studies led to more data to inform the model than in comparable studies (Lagarde et al., 2012), a wider range of data sources would have enabled more accurate model parameters and investigation of assumptions. For example, we did not have information to model the effect of expanded training on exit rates from Malawi and the public sector (i.e. cross-elasticities of the demand for training places) or the likely changes in doctors' preferences for training places over a 40-year period. Probabilistic sensitivity analysis will have accounted for this uncertainty to some degree. Finally, the effectiveness measures were derived from stated preferences and therefore can only be a guide to what doctors will accept in reality. For example, despite junior doctors indicating that they would accept five years of service in exchange for guaranteed specialty training, such a period may be difficult to implement and two years is likely to be more pragmatic.

With regard to specialty training, our reliance on tuition fees may have underestimated the true cost of providing training. Larger numbers of registrars are likely to be easily absorbed by the extensive medical educational system in South Africa, whereas more teaching staff would be required in Malawi (this would be the main associated cost as most specialties are taught via apprenticeship-style training in central hospitals). These posts are likely to be filled by expatriate doctors until sufficient Malawian specialists were trained, with expenses covered by the Malawian government and development partners. However, training was a small percentage of costs across all alternatives and therefore this is unlikely to be a substantive source of bias. For these purposes, we also disregarded concerns over the quality of specialty training that can be provided in Malawi without exposure to more advanced health systems. Similar concerns were raised when the medical school in Malawi was established and shown later to be without basis (Broadhead and Muula, 2002). Although we did not compare the expansion of training against purely financial interventions in this analysis, non-financial incentives are likely to be more tenable to the Malawian government in the short-term given the financial burden imposed by previous salary increases and current fiscal constraints (Mandeville and Muula, 2016).

In conclusion, this study has shown that expanding specialty training in Malawi is more cost-effective than training outside Malawi, despite being less valued by junior doctors. This policy direction will enlarge the specialist workforce substantially and obtain the most value from investment in undergraduate medical education, however will require increasing current spending levels. Implementation of this policy will need to take into account the existing and future skill mix of specialists in Malawi, as well as the likely need to incentivise unpopular but priority specialties such as ophthalmology (Mandeville et al., 2016b). The incentives offered to returning specialists will also need to be reviewed as the workforce expands, given that these accounted for up to a fifth of spending. The general conclusions from this study, rather than the specific ICERs, are likely to be transferable to other sub-Saharan African countries seeking to maximise the value from their investment in medical undergraduate education. Future research to refine the model would be welcomed, particularly in settings with more data available to clarify the assumptions employed here. Yet even in settings with less data, more routine application of cost-effectiveness analyses to health workforce decisions is likely to be of considerable value.

## Appendix A Supplementary data

Technical appendix Technical appendix

Model file Model file

## Footnotes

Appendix A Supplementary data related to this article can be found at https://doi.org/10.1016/j.socscimed.2017.10.012.
