# Supplementary material for: Specialty training for the retention of Malawian doctors: A cost-effectiveness analysis
Source: Soc Sci Med. 2017 Dec;194:87–95. doi: 10.1016/j.socscimed.2017.10.012 (PMC5710765; doi:10.1016/j.socscimed.2017.10.012)
Supplement: Technical appendix [file mmc1.pdf]

TECHNICAL APPENDIX

This document gives further details on the estimation of model parameters and threshold values. We also provide additional results first for the whole population and then the four subgroups.

MODEL PARAMETERS

To populate the model, four types of parameters are required: the model population, the probability of transitioning from one state to another, the uptake of training places and cost estimates. Details are provided on each in turn. The data sources to inform these parameter values are listed in Table A1.

Table A1 Data sources for model parameter values

PARAMETER	DATA SOURCE
**Model population**	
Cohort size	COM registry data
Existing stock	Ziljstra and Broadhead, 2007<br>Mandeville et al, 2014<br>Medical Council of Malawi 2010/2011 registry data
**Transition probabilities**	
Exit health labour market (*EHLM)*	Ziljstra and Broadhead, 2007<br>Lagarde and Cairns, 2012
Exit public sector	
Intern (EPS_I)	Mandeville et al, 2014
Medical officer (EPS_{MO})	Mandeville et al, 2014
Generalist (EPS_G)	Ziljstra and Broadhead, 2007
Exit Malawi	
Intern (EPS_I)	Mandeville et al, 2014
Medical officer (EM_{MO})	Mandeville et al, 2014
Generalist (EM_G)	Ziljstra and Broadhead, 2007
Specialist (EM_S)	COM data
**Uptake of training places**	
Baseline	Ministry of Health 15-year specialist forecast, COM data
Policy interventions	Mandeville et al, 2016
**Cost estimates**	
Salary	Ministry of Health, Malawi
Pension contributions	Pension Act 2011, Malawi
Accommodation	Southern, Northern and Central Regional Offices for Housing, Malawi
Communication	Key informant interviews
Transport	
Minibus	Key informant interviews
Specialist vehicle purchase	Key informant interviews
Monthly fuel subsidy	Malawi Energy Regulatory Authority
Postgraduate training costs	COM data, South African universities' websites

Model population

The existing stock of Malawian doctors was estimated from three sources: a tracing study of all graduates from the College of Medicine-University of Malawi (COM) between 1992 and 2006, another tracing study of all graduates between 2006 and 2012, and registration records for specialists from the Medical Council of Malawi for 2010/2011.

The 1992 - 2006 tracing study allowed an estimate of generalist doctors and their sector of work, along with doctors who had exited the health labour market (see below) (Zijlstra & Broadhead, 2007). The 2006 - 2012 tracing study identified all graduates in specialty training and their location (Mandeville et al., 2014). Registrars were assumed to be in their first year of training, unless they were in South Africa as part of sandwich training in which case they were assumed to be in their third year. All interns at the time of the 2006 – 2012 study entered the model in the medical officer state in order to accommodate the first cohort of new doctors in the model (see below). The registration records allowed a broad estimate of the number of Malawian specialists and their training location. All specialists who trained outside Malawi were placed in the South Africa specialist state unless recently trained in a specialty known to have sandwich training). Table A2 summarises these estimates across model states.

Table A2 Estimated existing Malawian doctors across model states in cycle 1

State	Number	State	Number
Intern	0	Malawi specialist	2
Medical officer	144	Sandwich specialist	2
Malawi registrar year 1	16	South Africa specialist	33
Sandwich registrar year 1	16	Generalist	107
South Africa registrar year 1	23	Exit Malawi	55

Transition probabilities

A matrix of all transition probabilities is shown in Table A3 and initial transition probability values and their distributions in Table A4. The transition probabilities from temporary to absorbing states will first be described, followed by those to specialty training.

Transition probabilities to absorbing states

The transition probabilities for the three absorbing states were constrained to be positive. For the first absorbing state, exit of the health labour market, the transition probability was assumed not to vary across states. As this state encompasses death, retirement, changing professions, and childrearing, the transition probability should increase with time, i.e.:

$$\text{EHLM}(t) = 1\text{-}e^{-\gamma t} \qquad (1)$$

Where t is cycle number and γ is the exit rate.

Table A3 Transition matrix

Notes: *PT = pre-training; EHLM = exit health labour market; SA = South Africa; *time-dependent parameter; §time- and cohort-dependent parameter*

ORIGIN	DESTINATION												
	**Intern**	**Medical officer**	**PT medical officer**	**Malawi registrar**	**Sandwich registrar**	**SA registrar**	**Malawi specialist**	**Sandwich specialist**	**SA specialist**	**Generalist**	**Exit Malawi**	**Exit public sector**	**EHLM**
**Intern**	0	1- EM_I -EPS_I –*EHLM*	0	0	0	0	0	0	0	0	EM_I	EPS_I	*EHLM**
**Medical officer**	0	1- *ET* - T_M - T_{MSA} - T_{SA} -EM_{MO} - EPS_{MO} – *EHLM*	ET_{M2-5} or ET_{MSA2-5} or ET_{SA2-5}	T_M or ET_M	T_{MSA} or ET_{MSA}	T_{SA} or ET_{SA}	0	0	0	G_{MO}*	EM_{MO}	EPS_{MO}	*EHLM**
**PT medical officer**	0	0	0	1 – *EHLM*	1 – *EHLM*	1 – *EHLM*	0	0	0	0	0	0	*EHLM**
**Malawi registrar**	0	0	0	0	0	0	1	0	0	0	0	0	0
**Sandwich registrar**	0	0	0	0	0	0	0	1	0	0	0	0	0
**SA registrar**	0	0	0	0	0	0	0	0	1	0	0	0	0
**Malawi specialist**	0	0	0	0	0	0	1- EM_M - *EHLM*	0	0	0	EM_M§	0	*EHLM**
**Sandwich specialist**	0	0	0	0	0	0	0	1- EM_{MSA} –*EHLM*	0	0	EM_{MSA}§	0	*EHLM**
**SA specialist**	0	0	0	0	0	0	0	0	1- EM_{SA} – *EHLM*	0	EM_{SA}§	0	*EHLM**
**Generalist**	0	0	0	0	0	0	0	0	0	1- EM_G - EPS_G – *EHLM*	EM_G*	EPS_G*	*EHLM**
**Exit Malawi**	0	0	0	0	0	0	0	0	0	0	1	0	0
**Exit public sector**	0	0	0	0	0	0	0	0	0	0	0	1	0
**EHLM**	0	0	0	0	0	0	0	0	0	0	0	0	1

Table A4 Initial transition probability values

Parameter description	Mean	Distribution	SE	α	B
Medical officer to generalist *(G_{MO})*	Cycle 8 = 1,<br>All other cycles = 0	Deterministic	-	-	-
Registrar to specialist	1	Deterministic	-	-	-
Specialty training uptake					
Training in Malawi *(T_M)*	0.02	Dirichlet	-	2	98.0
Training in Malawi and South Africa *(T_{MSA})*	0.08	Dirichlet	-	4	96.0
Training in South Africa *(T_{SA})*	0.08	Dirichlet	-	4	96.0
Medical officer to medical officer before training (*ET*)	Dependent on effectiveness measures	Dirichlet	-	-	-
Absorbing states					
Exit health labour market (*EHLM*)	$\gamma = 0.00175$*	Gamma[§]	0.001	0.175	99.8
Exit public sector					
Intern (EPS_I)	0.060	Dirichlet	-	6.0	97.2
Medical officer (EPS_{MO})	0.020	Dirichlet	-	2.0	97.2
Generalist (EPS_G)	0.02*, $\theta = 0.023$	Dirichlet	-	2.0	98.0
Exit Malawi					
Intern (EM_I)	$\zeta = 0.027$	Dirichlet	-	2.7	97.2
Medical officer (EM_{MO})	$\zeta = 0.027$	Dirichlet	-	2.7	97.2
Generalist (EM_G)	0.027*, $\theta = 0.023$	Dirichlet	-	2.7	97.3
Malawi-trained specialist (EM_M)	$\rho = 0.1$**, $\delta = 0.0023$	Dirichlet	-	3.0	97.0
Sandwich-trained specialist (EM_{MSA})	$\rho = 0.15$**, $\delta = 0.0023$	Dirichlet	-	10.0	90.0
South African-trained specialist (EM_{SA})	$\rho = 0.25$**, $\delta = 0.0023$	Dirichlet	-	16.7	83.3

Notes: *SE = standard error; *time-dependent; **time- and cohort-dependent, see below for values; [§]gamma rather than Dirichlet distribution assigned in order to more realistically contain the range of values obtained from the exponential function*

The 1992 – 2006 tracing study found that eight graduates had died and four were employed in Malawi but not in any mainstream health sector roles (Zijlstra & Broadhead, 2007). As no further detail was supplied, we assumed that these four graduates had exited the health labour market. This is likely an underestimate of the population rate, however, as the study participants were from successive and progressively younger cohorts and none had attained retirement age. We therefore used this rate (12 exits at 15 years) to anchor the lower limit for γ (-0.001). We based the upper limit (-0.0025) on the value used for a similar analysis of the nursing profession in South Africa, as exit for reasons of child- or eldercare in this female-dominated profession is likely to be higher than that of medicine in Malawi (Lagarde & Cairns, 2012). A mid-range value (-0.0175) between these two was used in the model, with uncertainty explored in the sensitivity analysis. Figure A1 shows the values of this parameter in each cycle.

Figure A1 Transition probabilities for "exit health labour market" parameter

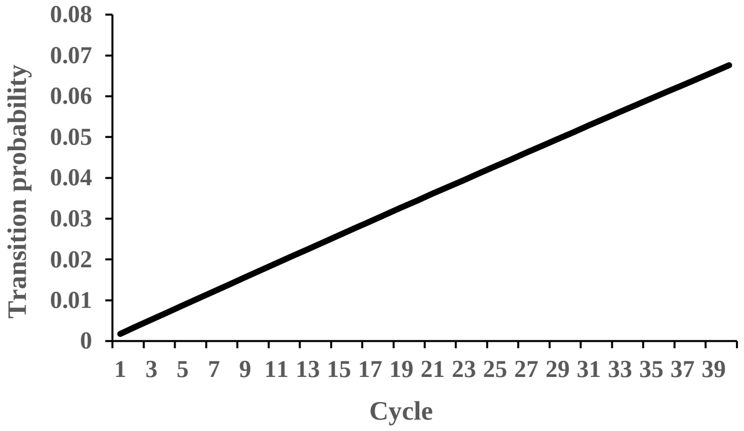

The transition probabilities for exiting the public sector for interns and medical officers was based the 2006 – 2012 tracing study (Mandeville et al., 2014). This allowed an examination of the proportion of doctors outside the public sector by time after graduation. This showed a polynomial rather than linear trend, with a steep rise for the first two years after graduation then a plateau (Figure A2). We used data on 2006 graduates outside the public sector to anchor transition probabilities for the intern and medical officer states.

Figure A2 Proportion of graduates outside public sector by year of graduation

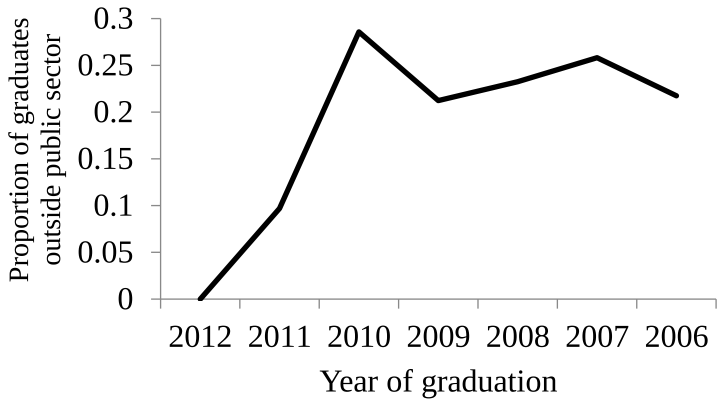

Source: *Mandeville et al, 2014.*

The probability of exiting Malawi for interns and medical officers was also based on examination of data from the 2006 – 2012 tracing study (Mandeville et al., 2014). In contrast, this showed a more linear trend for the proportion of doctors outside Malawi with time after graduation, even excluding those in government training programmes (Figure A3). Therefore, we used data on the proportion of 2006 graduates outside Malawi and not in government training programmes to anchor transition probabilities that reflected this trend.

Figure A3 Proportion of graduates outside public sector by year of graduation

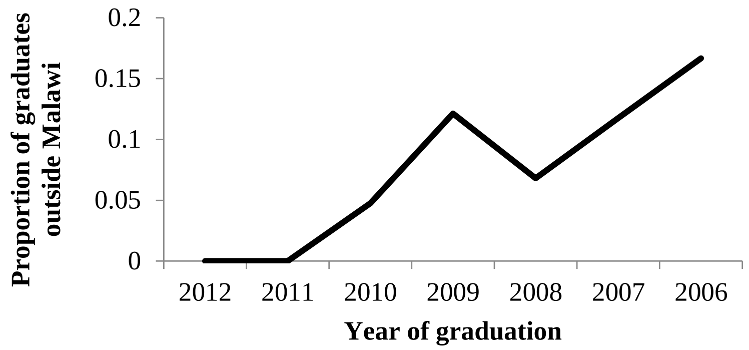

Source: Mandeville et al, 2014.

With regard to specialists, only the exit from Malawi is relevant (as it was assumed specialists did exit the public sector even if engaged in dual practice). From limited COM data on current posts of specialists trained in the last ten years, we estimated a probability, ρ, of exiting Malawi each cycle where ρ_M is for specialists who have trained all in Malawi, ρ_{SA} for specialists who have trained all in South Africa, and ρ_{MSA} for specialists with sandwich training. This value declined with time in order to reflect the likely "magnet effect" of an expanding specialist workforce (Bailey, Mandeville, Rhodes, Mipando, & Muula, 2012). Therefore, the functional form for exiting Malawi from each cycle in the specialist state was:

$$EM(t) = \rho + (1-e^{\delta t}) \quad (2)$$

with δ set so that the transition probability halved after 20 years for the first cohort. As the magnet effect would be greater for subsequent cohorts, we made this parameter both cohort- and time-dependent by setting ρ to decrease by 10% for each cohort until equal to the background exit probability for generalist doctors (see below). Figure A4 provides an overview of these transition probabilities over the time horizon for each specialist state.

In the generalist state, the exit probabilities for the medical officer state were used as the basis for a declining exponential function to model exits from Malawi and the public sector, such that:

$$EM_G(t) = 0.02 + (1-e^{\theta t}) \quad (3)$$

and:

$$EPS_G(t) = 0.027 + (1-e^{\theta t}) \quad (4)$$

Data from the earlier tracing study (Zijlstra & Broadhead, 2007) were used to inform the value of θ. We defined generalists as all graduates who were not specialists or in postgraduate training programmes. Out of 186 generalists, 34 (18.3%) were outside Malawi and 21 (11.3%) outside the public sector at a maximum 14 years of follow-up for the earliest graduates. Figure A5 shows the time-dependent values for these two parameters.

Figure A4 Transition probabilities over time horizon from specialist states to Exit Malawi

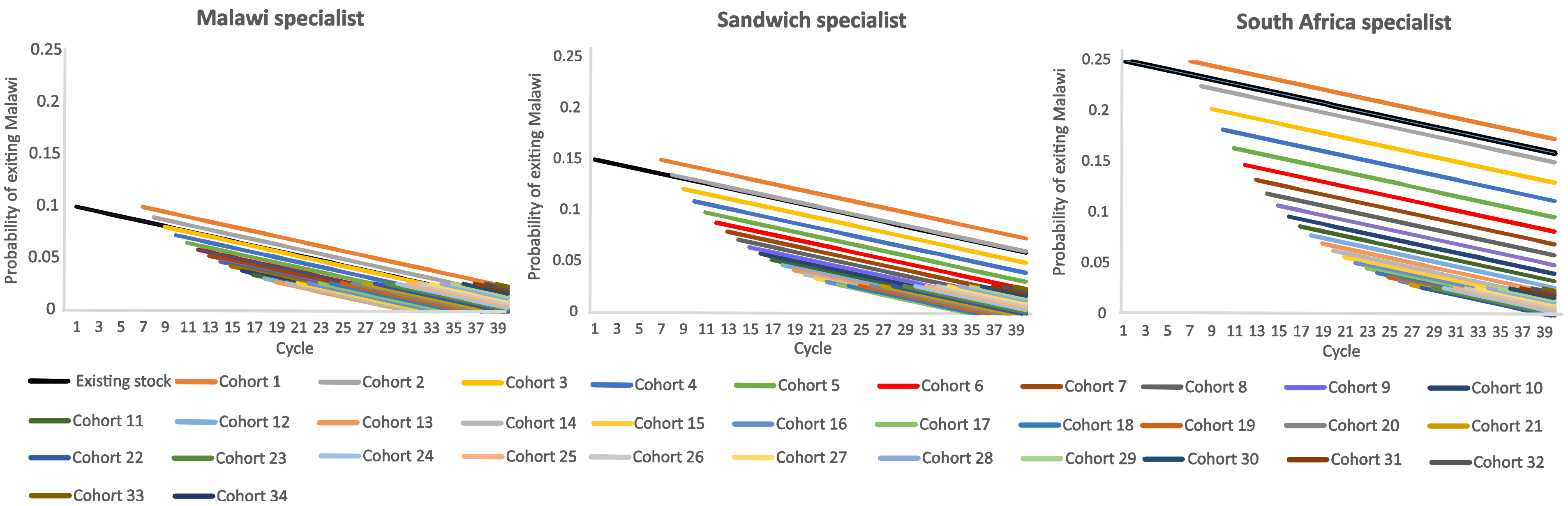

Note: *Cohorts 35 to 40 not shown as these cohorts do not reach specialist states by the end of the time horizon*

Figure A5 Time-dependent transition probabilities from generalist state

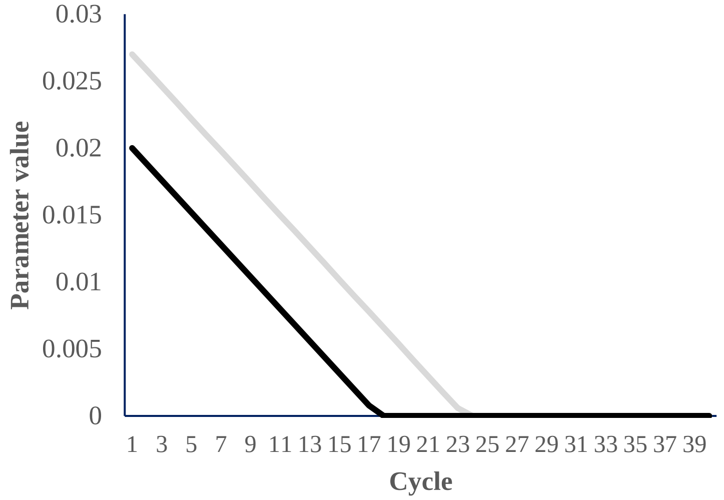

Notes: *Exit Malawi transition probability for generalists (EM_G) shown in grey and exit public sector transition probability for generalists (EPS_G) shown in black. Cycle numbers indicate the first cycle in which doctors enter generalist state.*

Transition probability from medical officer to registrar in baseline scenario

The probability of transitioning to specialty training in the baseline is challenging to estimate for several reasons: (i) there are multiple funders of training places in Malawi (including the government, Christian Health Association of Malawi (CHAM), research organisations and charities), but this analysis is focused on government funding; (ii) historically funding has been provided on an *ad hoc* basis due to budgetary constraints, therefore it is difficult to predict the annual number of available training place (whilst the MOH has forecast the minimum number of specialists required between 2009 and 2023 in order to maintain current specialist provision in central hospitals, this is not a budgetary commitment); (iii) there are important cohort effects, with a small stock of doctors in earlier years and greater number of graduates more recently.

Examining the tracing study dataset for the proportion of graduates in specialty training per year of graduation would be misleading for this reason. Moreover, many earlier registrars were funded by a grant from the National AIDS Commission (NAC), under which 34 registrars started between 2010 and 2012, however this funding has been discontinued.

In order to inform this transition probability, we constructed two forecasts for the funding of training places. In the 'replacement' scenario, the MOH 15-year forecast was compared to COM registry data on enrolled students by year in order to predict the percentage of medical officers that could start specialty training per year under the assumptions (i) the government funded training places based on this plan and (ii) all places were taken up (Table A5). This drops to 11% of interns by 2020. In the 'optimistic' scenario, we envisaged a similar budgetary commitment as the NAC grant. We compared the number of places offered per year under this grant compared to the number of predicted medical officers that year, finding that this represented around a quarter of new medical officers each year.

Table A5 **Comparison of specialty training data and predicted medical officer numbers**

Year	Predicted number of medical officers*	MOH 15-year forecast			NAC grant	
		Number of specialists Required	Specialty training places required**	Training places as percentage of interns	Number of NAC training places§	Training places as percentage of medical officers
**2009**	48	20	17	0.35	11	0.23
**2010**	38	18	17	0.45	11	0.29
**2011**	55	18	17	0.31	12	0.22
**2012**	53	17	15	0.28	-	-
**2013**	62	17	15	0.24	-	-
**2014**	53	17	14	0.26	-	-
**2015**	61	15	14	0.23	-	-
**2016**	80	15	14	0.18	-	-
**2017**	98	14	14	0.14	-	-
**2018**	104	14	13	0.13	-	-
**2019**	86	14	11	0.13	-	-
**2020**	99	14	11	0.11	-	-
**2021**	-	13	-	-	-	-
**2022**	-	11	-	-	-	-
**2023**	-	11	-	-	-	-

Notes: *Data from MOH and COM; *Based on number of graduates from previous academic year or medical students enrolled in relevant year, under assumption that all enrolled students graduate, complete internship and stay in the public sector; **Based on a lag of four years from the number of specialists required assuming a four-year training programme; §Places are allocated between years as planned rather than actual annual intake which was affected by implementation delays*

To elicit a more realistic forecast anchored by these best- and worst-case scenarios, we took the mean of the ratios of medical officer to training places for the three years of the NAC grant and the next five years of the replacement scenario. This gave an average of 18% of medical officers transitioning to registrars. We used the percentage of registrars in different training locations in the NAC grant to divide the transition probability between the three possible registrar states: 2% for Malawi training and 8% each for sandwich or South African training.

Transition probability from medical officer to registrar states with policy interventions

The probability of transitioning to specialty training under different policy interventions were based on a discrete choice experiment of junior doctors’ training preferences (Mandeville et al., 2016). Distinct subgroups of junior doctors with different preferences for specialty training were identified by this study. The uptake rates of different policy interventions were therefore calculated across all subgroups combined (weighted by the size of the subgroup in the overall sample) and then for each subgroup in turn. These training uptake rates are shown in Table A6.

Table A6 Predicted uptake of expanded training under policy interventions

Policy intervention	PREDICTED UPTAKE (%)				
	Weighted average	Rich rejecters	Stubborn specialists	Money motivated	Pliant patriots
Expanded Malawian training	0.54	0.19	0.50	0.56	0.83
Expanded sandwich training	0.68	0.40	0.65	0.70	0.89
Expanded South African training	0.70	0.42	0.68	0.78	0.88
Expanded Malawian training + 2 years	0.39	0.05	0.37	0.40	0.66
Expanded sandwich training + 2 years	0.52	0.13	0.52	0.56	0.76
Expanded South African training + 2 years	0.55	0.14	0.55	0.66	0.75
Expanded Malawian training + 3 years	0.32	0.02	0.31	0.33	0.55
Expanded sandwich training + 3 years	0.44	0.06	0.45	0.48	0.67
Expanded South African training + 3 years	0.47	0.07	0.48	0.58	0.65
Expanded Malawian training + 4 years	0.26	0.01	0.25	0.27	0.44
Expanded sandwich training + 4 years	0.37	0.03	0.38	0.41	0.56
Expanded South African training + 4 years	0.40	0.03	0.42	0.51	0.55
Expanded Malawian training + 5 years	0.20	0.01	0.20	0.21	0.33
Expanded sandwich training + 5 years	0.30	0.01	0.32	0.34	0.44
Expanded South African training + 5 years	0.33	0.02	0.35	0.43	0.43

Cost estimates

Further details are given here on the cost estimates used in the study, with all values and distributions shown in Table A7.

Salary

The latest public sector salary scales (October 2014) were obtained from the Ministry of Health. The entry-level monthly salary for an intern is MWK 321,560, which includes a basic salary of MWK 208,855 and several allowances specific to health workers and doctors. This equates to a net annual salary of MWK 2,782,104**.**

Four salary grades are relevant to this model: HH (entry level), HG, HF and HE. All have six points except HE which has four points. Doctors progress one salary point per year of service and are eligible for promotion to the next grade after four years, except for HE which is only obtained after completion of specialty training. As approval of promotions can be delayed (Chimwaza et al., 2014; Muula & Maseko, 2005), we stipulated that doctors moved up one salary point per cycle for all six salary points before moving to the next grade. We assume that doctors in CHAM facilities are paid at the same rate as those in MOH facilities. In this model, all doctors in the generalist state are promoted to HF grade, as this is the usual level for senior district doctors (Muula & Maseko, 2005). As it was only possible to distinguish specialists and generalists by year of service for the first four cycles in each state, salary costs for doctors entering these states in cycle 5 and above were set at the highest point.

While health workers in the public sector can earn extra income from overtime shifts, it is uncommon for doctors to undertake these as the remuneration is several times less than can be earned for equivalent shifts in private hospitals. Therefore no estimate for overtime was included in the costs.

Pension

The Pension Act of 2010 mandated pension contributions for all employers to a national pension scheme, set as a minimum of 10% of salary costs for employers and 5% for employees (Government of Malawi, 2011; Mhango & Thejane, 2012). Therefore, all salaries were uplifted by 10% to cover employer contributions.

Accommodation

All public sector doctors are entitled to government accommodation. This may be owned by the Ministry of Health, Ministry of Lands and Housing or rented from either private landlords or the Malawi Housing Corporation (a statutory body). As data on capital investment and maintenance for government houses were not available, we used rental costs instead. Although interns are entitled to accommodation in hospital owned flats, the capacity is inadequate for the growing number of graduates and many interns are provided with rented accommodation. Data on current rents paid for doctors working in central and district hospitals were obtained from the three Regional Offices for Housing in Malawi. Although caps for government contributions to rented accommodation have been set across the public sector, the housing data showed that these caps were regularly breached in the major cities.

Accommodation continues to be subsidised while registrars are training in South Africa. Although immediate family would continue to be eligible for free accommodation whilst a registrar is in South Africa, most junior doctors were unmarried and without children in the DCE dataset. Therefore Malawian accommodation costs were not counted during training in South Africa, although residency fees for registrars were included in tuition fee estimates.

Transport

There is a one-off transport incentive associated with the transition to a specialist. This is a recent implemented policy whereby the government purchases a vehicle for each newly appointed specialist in the public sector. The vehicle is then owned by the specialist, who bears all responsibility for maintenance. The vehicle is a standard saloon car bought from Malawi-based dealers, with an estimated cost of MWK10 million. The government also provides 250 litres of fuel per month for all specialists. An average of the Malawian Energy Regulatory Authority recommended pump prices for petrol and diesel for May 2015 were used in these estimates (Malawi Energy Regulatory Authority).

The other transport subsidy is for interns and medical officers at central hospitals, who should have transport to and from work provided by the hospital. This is usually in the form of a shared minibus with other hospital staff. As the cost per doctor was therefore difficult to estimate, we instead took the market price of a journey using a public shared minibus. Junior doctors are usually accommodated relatively far from the central hospitals due to the rental caps, therefore we used an average of estimated cost of journeys to these areas in Lilongwe and Blantyre. As doctors based at district hospitals have access to hospital vehicles for personal transport, we used the same cost estimate per journey for all junior doctors.

Communication costs

All specialists in Malawi receive a standard monthly allowance to cover landline and mobile phone costs, which were included in the estimates.

Specialty training allowances

Although registrars continue to receive their MOH salary (and increments) during training, they also receive an additional stipend (higher during South African training to cover increased living costs). Registrars also receive annual conference and book allowances, along with a one-off entry cost of a laptop. Registrars in South Africa also receive allowances to cover health insurance and costs related to clinical work, e.g. transport to clinics. There is also a one-off settling-in allowance at the start of South African training, and baggage allowances on entry and exit.

Table A7 Cost estimates

Description	Mean cost (MWK)	Units	Annual cost	SE	Distribution	α	β
**Gross monthly salary**							
HH grade							
Point 1	321,560	12	3,858,720	-	Deterministic	-	-
Point 2	327,658	12	3,931,896	-		-	-
Point 3	333,756	12	4,005,072	-		-	-
Point 4	339,854	12	4,078,248	-		-	-
Point 5	345,953	12	4,151,436	-		-	-
Point 6	352,051	12	4,224,612	-		-	-
HG grade							
Point 1	359,609	12	4,315,308	-	Deterministic	-	-
Point 2	372,721	12	4,472,652	-		-	-
Point 3	385,832	12	4,629,984	-		-	-
Point 4	398,944	12	4,787,328	-		-	-
Point 5	412,055	12	4,944,660	-		-	-
Point 6	425,167	12	5,102,004	-		-	-
HF grade							
Point 1	458,371	12	5,500,452	-	Deterministic	-	-
Point 2	499,922	12	5,999,064	-		-	-
Point 3	541,473	12	6,497,676	-		-	-
Point 4	583,023	12	6,996,276	-		-	-
Point 5	624,574	12	7,494,888	-		-	-
Point 6	666,125	12	7,993,500	-		-	-
HE grade							
Point 1	845,070	12	10,140,840	-		-	-
Point 2	884,515	12	10,614,180	-		-	-
Point 3	923,961	12	11,087,532	-		-	-
Point 4	963,406	12	11,560,872	-		-	-
**Pension**							
Employer contributions	10% of salary				Deterministic	-	-
**Accommodation**	102,080	12	1224955	93,959	Gamma	170	7,207
**Transport**							
Interns and medical officers							
Minibus journey to and from work	250	2 per day	182,500	70000	Gamma	7	26,849
Specialists							
Vehicle purchase	10,000,000	One-off	-	1,000,000	Gamma	100	100,000
Monthly fuel subsidy	729	250 litres	2,187,000	218,700	Gamma	100	21,870
**Communications (specialists only)**							
Mobile and landline allowance	36,000	12	432,000	43200	Gamma	100	4,320
**Specialty training**							
All registrars							
Laptop	572,727	1	572,727	57273	Gamma	100	5,727
Book allowance	211,909	1	211,909	21191	Gamma	100	2,119

Conference allowance	257,729	1	257,729	25773	Gamma	100	2,577
Registrars in Malawi							
Stipend	2,646,000	1	2,646,000	264600	Gamma	100	26,460
Tuition fees	550,000	1	550,000	55000	Gamma	100	5,500
Registrars in South Africa							
Tuition fees	2,332,153	1	2,332,153	233215	Gamma	100	23,322
Stipend	4,467,273	1	4,467,273	446727	Gamma	100	44,673
Clinical work allowance	1,338,876	1	1,338,876	133888	Gamma	100	13,389
Health insurance	343,636	1	343,636	34364	Gamma	100	3,436
Settling in allowance	211,909	One-off	-	21191	Gamma	100	2,119
Baggage allowance	114,545	Entry & exit	-	11455	Gamma	100	1,145

Notes: *MWK = Malawian kwacha; SE = standard error (derived from sample for accommodation, 10% of point estimates for other values)*

THRESHOLD VALUE BY OUTCOME MEASURE

In order to identify the threshold that the Malawian government is currently willing to pay for a doctor- and specialist-year, we calculated the net present value of the discounted costs of an "average" doctor working for 40 years in the public sector.

This incorporated two career pathways: a "generalist path" in which a medical officer becomes a generalist after six years and a "specialist path" where specialty training is entered after one year as a medical officer. These were weighted 0.82 and 0.18 respectively according to the forecast provision of government-funded specialty training in Table A5.

For a specialist-year, only the costs of the specialist path were included, with weighting of costs for different training locations as per the allocations used previously (0.11 for Malawi training and 0.44 for sandwich and South African training).

RESULTS: WHOLE POPULATION

Average costs and effects

Table A8 shows the mean values obtained from 2000 Monte Carlo simulations for doctor-years, specialist-years and costs, as well as the proportion of baseline these represent for each policy intervention.

Table A9 breaks down total costs for baseline and all interventions by cost category.

Incremental cost-effectiveness ratios (ICER)

The incremental effects, costs and ICERs for all interventions are shown in Table A10. An ICER represent the difference between the average costs of the intervention compared to baseline divided by the difference in their average effects. When interventions are ranked by incremental costs, it is possible to assess dominance and extended dominance. Interventions dominate the baseline if they are more effective (i.e. produce more doctor-or specialist-years), but are dominated by other interventions that are more effective and less costly. Extended dominance occurs where an intervention is less costly than another, but has a higher ICER.

If the policy aim is to maximise doctor-years, then the most cost-effective policy is expansion of training in Malawi with five years of mandatory service. This provides the most value at MWK19,411,755 per doctor-year, with shorter periods of service (4 and 2 years) being the next most cost-effective options. Expanding sandwich training confers the greatest increase in doctor-years over baseline, however at the highest ICER.

If the policy aim, however, is to maximise the value from specialty training, then just two years of mandatory service before training Malawi is the most cost-effective option at a cost of MWK10,099,894 per specialist-year. Expanding sandwich training is the only other cost-effective option, conferring the greatest increase in specialty-years over baseline, but with a higher ICER than training all in Malawi. As there are few specialists currently in Malawi, the incremental effects of any expansion of specialty training in terms of specialist-years is much higher than for doctor-years, leading to lower ICERs in general.

RESULTS: SUBGROUPS

Table A11 shows the mean values for doctor-years, specialist-years and costs for all subgroups.

Tables A12 to A15 then give the incremental effects, costs and ICERs for all interventions by subgroup.

Figure A6 shows the incremental cost-effectiveness acceptability frontiers (CEAF) for the subgroups not shown in the main text.

Table A8 Average costs and effects for whole population

Policy intervention*	Doctor-years		Specialist-years		Cost (MWK)	
	Mean	Proportion of baseline	Mean	Proportion of baseline	Mean	Proportion of baseline
Baseline	39,199	-	2,554	-	317,768,773,628	-
Expanded Malawi training	42,136	1.07	15,080	5.90	470,945,259,868	1.48
Expanded sandwich training	46,054	1.17	15,729	6.16	501,376,270,453	1.58
Expanded South Africa training	30,654	0.78	14,801	5.80	491,870,072,661	1.55
Expanded Malawi training + 2 years	42,084	1.07	10,114	3.96	394,120,512,350	1.24
Expanded sandwich training + 2 years	39,455	1.01	11,425	4.47	439,743,153,629	1.38
Expanded South Africa training + 2 years	34,876	0.89	10,506	4.11	441,245,319,782	1.39
Expanded Malawi training + 3 years	41,895	1.07	8,215	3.22	395,096,582,105	1.24
Expanded sandwich training + 3 years	40,072	1.02	9,310	3.65	423,107,762,965	1.33
Expanded South Africa training + 3 years	36,503	0.93	8,659	3.39	421,649,883,540	1.33
Expanded Malawi training + 4 years	41,624	1.06	6,706	2.63	373,534,841,239	1.18
Expanded sandwich training + 4 years	40,457	1.03	7,602	2.98	382,808,336,799	1.20
Expanded South Africa training + 4 years	37,715	0.96	7,191	2.82	392,336,819,531	1.23
Expanded Malawi training + 5 years	41,215	1.05	5,365	2.10	356,908,448,028	1.12
Expanded sandwich training + 5 years	40,627	1.04	6,111	2.39	372,229,530,576	1.17
Expanded South Africa training + 5 years	38,638	0.99	5,904	2.31	375,141,596,982	1.18

Notes: *MWK = Malawian kwacha; Mean obtained from 2000 Monte Carlo simulations; Years indicate length of mandatory service*

Table A9 Breakdown of total costs by cost category

Policy intervention	Percentage of total costs by cost category				
	**Salary**	**Accommodation**	**Training**	**Transport**	**Specialist perks***
Baseline	74.34	13.86	2.77	1.99	7.04
Expanded Malawi training	63.39	10.27	6.84	0.82	18.68
Expanded sandwich training	61.92	11.07	7.68	0.71	18.62
Expanded South Africa training	64.74	7.41	8.02	0.70	19.13
Expanded Malawi training + 2 years	74.26	12.15	2.03	1.38	10.18
Expanded sandwich training + 2 years	74.26	12.15	2.03	1.38	10.18
Expanded South Africa training + 2 years	78.23	10.56	1.20	1.27	8.75
Expanded Malawi training + 3 years	75.39	12.09	2.03	1.52	8.98
Expanded sandwich training + 3 years	78.31	11.14	1.24	1.49	7.81
Expanded South Africa training + 3 years	79.51	10.26	1.25	1.48	7.50
Expanded Malawi training + 4 years	75.06	12.65	2.14	1.70	8.45
Expanded sandwich training + 4 years	76.97	12.40	1.37	1.77	7.48
Expanded South Africa training + 4 years	78.48	11.38	1.34	1.72	7.08
Expanded Malawi training + 5 years	74.96	13.08	2.23	1.84	7.90
Expanded sandwich training + 5 years	77.23	12.79	1.41	1.92	6.65
Expanded South Africa training + 5 years	77.99	12.18	1.40	1.90	6.52

Notes: **Aggregates costs of communication and fuel allowances and vehicle purchase.*

Table A10 Incremental costs, effects and cost-effectiveness ratios for whole population

Policy intervention*		Doctor-years		Specialist-years	
	Incremental costs (MWK)	Incremental effects	ICER	Incremental effects	ICER
Expanded Malawi training + 5 years	39,139,674,400	2,016	19,411,755	2,812	13,920,719
Expanded sandwich training + 5 years	54,460,756,948	1,428	38,147,823	3,558	15,308,569
Expanded Malawi training + 4 years	55,766,067,611	2,425	22,995,148	4,152	13,431,691
Expanded South Africa training + 5 years	57,372,823,355	-561	-102,318,924	3,350	17,124,108
Expanded sandwich training + 4 years	65,039,563,171	1,258	51,712,011	5,048	12,883,539
Expanded South Africa training + 4 years	74,568,045,903	-1,484	-50,244,813	4,637	16,081,135
Expanded Malawi training + 2 years	76,351,738,722	2,885	26,462,379	7,560	10,099,894
Expanded Malawi training + 3 years	77,327,808,477	2,696	28,684,513	5,661	13,660,451
Expanded South Africa training + 3 years	103,881,109,912	-2,696	-38,535,741	6,105	17,015,522
Expanded sandwich training + 3 years	105,338,989,337	873	120,710,070	6,756	15,591,149
Expanded sandwich training + 2 years	121,974,380,001	256	476,121,939	8,871	13,750,308
Expanded South Africa training + 2 years	123,476,546,154	-4,323	-28,562,180	7,953	15,526,637
Expanded Malawi training	153,176,486,240	2,937	52,159,069	12,526	12,229,035
Expanded South Africa training	174,101,299,033	-8,545	-20,375,049	12,247	14,215,318
Expanded sandwich training	183,607,496,825	6,855	26,783,976	13,175	13,936,156

Notes: *Increments are over baseline values (see Table A8). Shading indicates dominated interventions with regard to doctor-years and specialists-years respectively. ICER = Incremental cost-effectiveness ratios; MWK = Malawian kwacha; *Years indicate length of mandatory service*

Table A11 Average costs and effects by subgroup

Policy intervention	Doctor-years				Specialist-years				Costs (MWK billion)			
	RR	SS	MM	PP	RR	SS	MM	PP	RR	SS	MM	PP
Baseline	39,270	39,211	39,155	39,193	2,567	2,574	2,598	2,578	318.73	318.35	318.07	318.20
Expanded Malawi training	40,232	41,928	42,221	43,705	6,693	14,126	15,582	21,975	369.15	459.57	477.13	554.95
Expanded sandwich training	43,019	45,740	46,293	48,347	9,653	15,086	16,164	20,286	422.23	493.09	507.53	561.19
Expanded South Africa training	34,317	30,917	29,650	28,322	9,647	14,402	16,341	18,117	419.14	486.14	514.06	538.64
Expanded Malawi training + 2 years	39,551	41,969	42,155	44,139	3,143	9,797	10,407	15,722	306.52	390.33	397.78	465.04
Expanded sandwich training + 2 years	39,418	39,478	39,451	39,499	4,122	11,472	12,211	15,914	340.47	440.57	450.58	500.99
Expanded South Africa training + 2 years	38,539	34,883	33,936	33,121	4,055	10,497	12,300	13,651	343.56	441.23	468.79	488.99
Expanded Malawi training + 3 years	39,333	41,829	41,961	43,883	2,468	8,059	8,466	12,632	330.49	393.61	397.88	445.31
Expanded sandwich training + 3 years	39,480	40,100	40,113	40,439	2,728	9,485	10,029	13,300	334.16	425.61	432.80	477.25
Expanded South Africa training + 3 years	39,301	36,428	35,752	35,260	2,850	8,817	10,308	11,288	336.24	424.09	446.08	460.41
Expanded Malawi training + 4 years	39,258	41,547	41,705	43,357	2,270	6,564	6,941	9,918	315.28	371.91	376.53	416.22
Expanded sandwich training + 4 years	39,474	40,489	40,557	41,021	2,179	7,772	8,296	10,672	310.32	385.17	392.09	424.15
Expanded South Africa training + 4 years	39,634	37,619	37,171	36,959	2,236	7,454	8,713	9,203	314.53	396.65	416.46	424.09
Expanded Malawi training + 5 years	39,271	41,239	41,295	42,594	2,262	5,408	5,575	7,530	315.23	357.71	359.60	386.55
Expanded sandwich training + 5 years	39,427	40,734	40,775	41,219	1,845	6,447	6,750	8,208	309.50	377.42	381.54	403.31
Expanded South Africa training + 5 years	39,721	38,586	38,314	38,316	2,071	6,132	7,194	7,133	312.22	379.10	396.53	395.50

Notes: *MWK = Malawian kwacha; RR= rich rejecters; SS = stubborn specialists; MM = money motivated; PP = pliant patriot; Mean obtained from 2000 Monte Carlo simulations;*

Table A12 Incremental costs, effects and cost-effectiveness ratios for rich rejecters subgroup

Policy intervention	Incremental costs (MWK)	Doctor-years		Specialist-years	
		Incremental effects	ICER	Incremental effects	ICER
Expanded Malawi training + 2 years	-12,201,954,377	281	-43,436,832	576	-21,191,915
Expanded sandwich training + 5 years	-9,227,506,138	157	-58,907,758	-721	12,791,251
Expanded sandwich training + 4 years	-8,410,571,271	204	-41,234,324	-388	21,700,925
Expanded South Africa training + 5 years	-6,506,306,832	451	-14,417,119	-496	13,115,268
Expanded South Africa training + 4 years	-4,200,441,411	364	-11,546,659	-331	12,686,170
Expanded Malawi training + 5 years	-3,492,568,125	1	-4,350,743,687	-305	11,439,714
Expanded Malawi training + 4 years	-3,450,521,624	-12	277,158,078	-297	11,626,881
Expanded Malawi training + 3 years	11,763,672,786	63	185,727,619	-99	-118,561,899
Expanded sandwich training + 3 years	15,428,456,607	210	73,543,640	161	95,721,812
Expanded South Africa training + 3 years	17,509,741,279	31	564,018,939	283	61,807,099
Expanded sandwich training + 2 years	21,741,826,905	148	146,898,683	1,555	13,981,971
Expanded South Africa training + 2 years	24,837,802,515	-731	-33,998,056	1,488	16,690,289
Expanded Malawi training	50,423,531,270	962	52,400,402	4,127	12,219,150
Expanded South Africa training	100,411,956,468	-4,953	-20,271,532	7,081	14,181,334
Expanded sandwich training	103,503,144,705	3,749	27,606,755	7,086	14,606,300

Notes: *ICER = Incremental cost-effectiveness ratios; MWK = Malawian kwacha. Increments are over baseline values (see Table A11). Shading indicates dominated interventions with regard to doctor-years and specialists-years respectively.*

Table A13 Incremental costs, effects and cost-effectiveness ratios for stubborn specialists subgroup

Policy intervention*	Incremental costs (MWK)	Doctor-years		Specialist-years	
		Incremental effects	ICER	Incremental effects	ICER
Expanded Malawi training + 5 years	39,361,528,692	2,028	19,411,459	2,833	13,891,941
Expanded Malawi training + 4 years	53,559,419,888	2,336	22,928,224	3,990	13,424,569
Expanded sandwich training + 5 years	59,065,846,167	1,522	38,800,053	3,873	15,251,700
Expanded South Africa training + 5 years	60,744,012,445	-625	-97,226,084	3,558	17,073,259
Expanded sandwich training + 4 years	66,819,821,043	1,278	52,304,632	5,198	12,855,257
Expanded Malawi training + 2 years	71,982,278,086	2,758	26,101,937	7,223	9,965,429
Expanded Malawi training + 3 years	75,254,410,694	2,618	28,748,696	5,485	13,719,609
Expanded South Africa training + 4 years	78,293,386,662	-1,592	-49,173,808	4,879	16,045,709
Expanded South Africa training + 3 years	105,741,886,458	-2,783	-37,997,620	6,243	16,937,499
Expanded sandwich training + 3 years	107,260,453,210	889	120,634,085	6,911	15,520,251
Expanded sandwich training + 2 years	122,218,357,667	267	457,958,240	8,898	13,734,953
Expanded South Africa training + 2 years	122,874,602,680	-4,328	-28,388,515	7,923	15,508,466
Expanded Malawi training	141,218,112,952	2,717	51,983,269	11,552	12,224,501
Expanded South Africa training	167,788,301,228	-8,294	-20,230,631	11,828	14,185,575
Expanded sandwich training	174,733,628,066	6,528	26,765,457	12,512	13,965,592

Notes: *ICER = Incremental cost-effectiveness ratios; MWK = Malawian kwacha. Increments are over baseline values (see Table A11). Shading indicates dominated interventions with regard to doctor-years and specialists-years respectively.*

Table A14 Incremental costs, effects and cost-effectiveness ratios for money motivated subgroup

Policy intervention*	Incremental costs (MWK)	Doctor-years		Specialist-years	
		Incremental effects	ICER	Incremental effects	ICER
Expanded Malawi training + 5 years	41,529,115,712	2,141	19,400,221	2,977	13,951,014
Expanded Malawi training + 4 years	58,463,029,846	2,550	22,928,043	4,343	13,461,268
Expanded sandwich training + 5 years	63,475,044,352	1,620	39,184,903	4,152	15,287,312
Expanded sandwich training + 4 years	74,021,890,100	1,402	52,805,480	5,698	12,990,060
Expanded South Africa training + 5 years	78,457,912,421	-841	-93,315,172	4,596	17,069,198
Expanded Malawi training + 2 years	79,712,550,090	3,000	26,569,959	7,809	10,207,268
Expanded Malawi training + 3 years	79,810,760,370	2,806	28,438,442	5,869	13,599,844
Expanded South Africa training + 4 years	98,395,881,219	-1,983	-49,609,229	6,115	16,090,526
Expanded sandwich training + 3 years	114,733,632,347	958	119,749,683	7,431	15,440,196
Expanded South Africa training + 3 years	128,016,663,014	-3,402	-37,625,499	7,710	16,603,681
Expanded sandwich training + 2 years	132,516,279,540	296	447,000,888	9,614	13,784,070
Expanded South Africa training + 2 years	150,724,077,778	-5,219	-28,882,092	9,702	15,535,627
Expanded Malawi training	159,064,905,022	3,066	51,872,248	12,984	12,250,728
Expanded sandwich training	189,459,875,358	7,139	26,540,171	13,566	13,965,967
Expanded South Africa training	195,992,295,124	-9,505	-20,619,856	13,743	14,261,188

Notes: *Increments are over baseline values (see Table A8). Shading indicates dominated interventions with regard to doctor-years and specialists-years respectively. ICER = Incremental cost-effectiveness ratios; MWK = Malawian kwacha; *Years indicate length of mandatory service*

Table A15 Incremental costs, effects and cost-effectiveness ratios for pliant patriots subgroup

Policy intervention*	Incremental costs (MWK)	Doctor-years		Specialist-years	
		Incremental effects	ICER	Incremental effects	ICER
Expanded Malawi training + 5 years	68,342,613,445	3,401	20,092,977	4,952	13,801,003
Expanded South Africa training + 5 years	77,292,490,052	-877	-88,105,004	4,555	16,970,505
Expanded sandwich training + 5 years	85,107,689,471	2,026	42,007,828	5,630	15,117,971
Expanded Malawi training + 4 years	98,011,998,346	4,164	23,536,068	7,340	13,353,340
Expanded South Africa training + 4 years	105,885,746,195	-2,234	-47,405,214	6,625	15,983,035
Expanded sandwich training + 4 years	105,945,891,573	1,828	57,942,585	8,094	13,088,885
Expanded Malawi training + 3 years	127,111,080,122	4,691	27,099,445	10,054	12,642,871
Expanded South Africa training + 3 years	142,207,033,928	-3,933	-36,155,218	8,710	16,326,722
Expanded Malawi training + 2 years	146,831,753,449	4,946	29,687,698	13,144	11,170,824
Expanded sandwich training + 3 years	159,048,815,400	1,246	127,628,490	10,722	14,834,479
Expanded South Africa training + 2 years	170,781,399,153	-6,072	-28,126,591	11,073	15,423,653
Expanded sandwich training + 2 years	182,789,444,184	306	598,007,848	13,336	13,706,387
Expanded South Africa training	220,432,506,955	-10,871	-20,277,226	15,538	14,186,271
Expanded Malawi training	236,746,290,079	4,512	52,471,246	19,397	12,205,097
Expanded sandwich training	242,991,089,056	9,154	26,544,706	17,708	13,722,293

Notes: *Increments are over baseline values (see Table A8). Shading indicates dominated interventions with regard to doctor-years and specialists-years respectively. ICER = Incremental cost-effectiveness ratios; MWK = Malawian kwacha; *Years indicate length of mandatory service*

Figure A6 Cost-effectiveness acceptability frontiers by subgroup

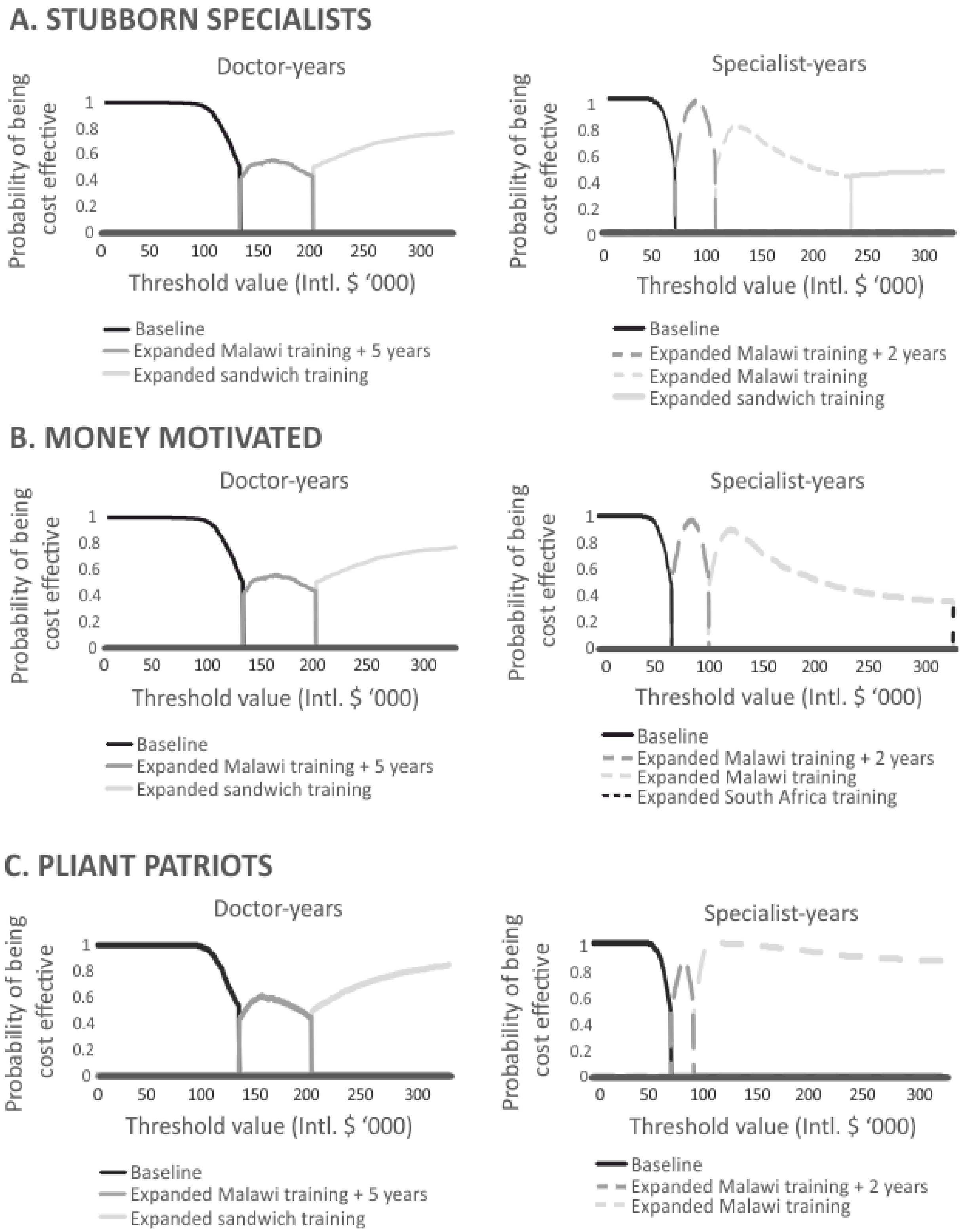

REFERENCES

Bailey, N., Mandeville, K. L., Rhodes, T., Mipando, M., & Muula, A. (2012). Postgraduate career intentions of medical students and recent graduates in Malawi: a qualitative interview study. *BMC Medical Education, 12*, 87. doi:10.1186/1472-6920-12-87

Chimwaza, W., Chipeta, E., Ngwira, A., Kamwendo, F., Taulo, F., Bradley, S., & McAuliffe, E. (2014). What makes staff consider leaving the health service in Malawi? *Human Resources for Health*, *12*(1), 17.

Government of Malawi. (2011). Pension Act 2010. *The Malawi Gazette Supplement 8 April 2011*. Available at: https://www.rbm.mw/documents/pisu/Pension%20Act%202011.pdf

Lagarde, M., & Cairns, J. (2012). Modelling human resources policies with Markov models: an illustration with the South African nursing labour market. *Health Care Management Science*, *15*, 270-282.

Malawi Energy Regulatory Authority. Press release: Fuel price adjustment for May 2015. Accessed 12 May 2015. Available at: http://www.meramalawi.mw.

Mandeville, K. L., Ulaya, G., Lagarde, M., Gwesele, L., Dzowela, T., Hanson, K., & Muula, A. (2014). Early career retention of Malawian medical graduates: a retrospective cohort study. *Tropical Medicine & International Health, 20*(1), 106-114. doi:10.1111/tmi.12408

Mandeville, K. L., Ulaya, G., Lagarde, M., Muula, A. S., Dzowela, T., & Hanson, K. (2016). The use of specialty training to retain doctors in Malawi: A discrete choice experiment. *Social Science & Medicine*, *169*, 109-118. doi:10.1016/j.socscimed.2016.09.034

Mhango, M., & Thejane, P. (2012). The Malawi Pension Act: A General Commentary on Some of its Core Mandatory Provisions with Specific Reference to Sections 9, 10 and 15. *South African Law Journal, 129*, 773-802.

Muula, A., & Maseko, F. C. (2005). *Survival and retention strategies for Malawian health professionals. EQUINET Discussion Paper No. 32*. Harare: EQUINET (Regional Network of Equity and Health in East and Southern Africa). http://www.equinetafrica.org/bibl/docs/DIS32HRmuula.pdf

Zijlstra, E., & Broadhead, R. (2007). The College of Medicine in the Republic of Malawi: towards sustainable staff development. *Human Resources for Health, 5*, 10. doi:10.1186/1478-4491-5-10
